# Changes in Phytochemical Synthesis, Chalcone Synthase Activity and Pharmaceutical Qualities of Sabah Snake Grass (*Clinacanthus nutans* L.) in Relation to Plant Age

**DOI:** 10.3390/molecules191117632

**Published:** 2014-10-30

**Authors:** Ali Ghasemzadeh, Alireza Nasiri, Hawa Z. E. Jaafar, Ali Baghdadi, Izham Ahmad

**Affiliations:** Department of Crop Science, Faculty of Agriculture, University Putra Malaysia, 43400 Serdang, Malaysia; E-Mails: a.nasiri1362@gmail.com (A.N.); Hawazej@upm.edu.my (H.Z.E.J.); ali_baghdadi@yahoo.com (A.B.); iz_ahmad@upm.edu.my (I.A.)

**Keywords:** sabah snake grass, flavonoid, chalcone synthase, DPPH, FRAP, HeLa cancer

## Abstract

In the current study, changes in secondary metabolite synthesis and the pharmaceutical quality of sabah snake grass leaves and buds were considered in relation to plant age (1 month, 6 months, and 1 year old). The activity of the enzyme chalcone synthase (CHS, EC 2.3.1.74) was measured, as it is a key enzyme for flavonoid production. Significant differences in total flavonoid (TF) production were observed between the three plant growth periods and the different plant parts. The highest contents of TF (6.32 mg/g dry weight [DW]) and total phenolic (TP) (18.21 mg/g DW) were recorded in 6-month-old buds. Among the flavonoids isolated in this study the most important ones based on concentration were from high to low as follows: catechin > quercetin > kaempferol > luteolin. Production of phenolic acids increased from 1 to 6 months, but after 6 months up to 1 year of age, they decreased significantly. The highest contents of caffeic acid (0.307 mg/g DW) and gallic acid (5.96 mg/g DW) were recorded in 1-year and 6-month-old buds, respectively. The lowest and highest activity of CHS was recorded in 1-month and 6-month-old buds with values of 3.6 and 9.5 nkat/mg protein, respectively. These results indicate that the increment in flavonoids and phenolic acids in 6-month-old buds can be attributed to an increase in CHS activity. The highest 1,1-diphenyl-2-picrylhydrazyl (DPPH) activity was observed in the extract of 1-year-old buds followed by 6-month-old buds, with 50% of free radical scavenging (IC_50_) values of 64.6 and 73.5 µg/mL, respectively. Interestingly, a ferric reducing antioxidant power (FRAP) assay showed a higher activity in 6-month-old buds (488 μM of Fe(II)/g) than in 1-year-old buds (453 μM of Fe(II)/g), in contrast to the DPPH result. Significant correlations (*p* < 0.05) were observed between CHS enzyme activity and FRAP activity, TF, catechin, and kaempferol content. Extracts of 6-month-old bud exhibited a significant *in vitro* anticancer activity against HeLa cancer cells with IC_50_ value of 56.8 µg/mL. These results indicate that early harvesting of snake grass (6-month-old) may yield increased concentrations of secondary metabolites, which are potent antioxidant compounds.

## 1. Introduction

As a fine source of bioactive compounds, medicinal plants have long been utilized both in traditional and modern medicine as nutraceuticals as well as food supplements. They are also used as pharmaceutical intermediates and chemical antecedents for synthetic drugs. The World Health Organization (WHO) has announced that in the developing countries, 80% of people utilize traditional medicines for their primary health care, while also noting that 85% of such medicines in these regions are derived from plant extracts [1]. Some of the phytochemicals that are extracted from plant sources include phenolics, flavonoids, alkaloids, saponins, tannins, and lignin. In practice, such materials are recognized as having the capability to prevent, as well as treat, various health problems such as cancer, heart disease, diabetes, and high blood pressure [2].

Today, a valuable line of study in the medical and food industry is appraising phytochemicals to ascertain whether they have biological activities with potential benefits to human health [3]. Recently, there has been a discernible increase in the utilization of plants as a source of natural antioxidants in order to scavenge free radicals [4,5,6]. Particular factors can affect the flavonoid content in the herbs and medicinal plants. These factors include the environmental conditions, year and season, along with the plant’s age, leaf maturity, and similar growth factors. It has been found by Fritz* et al.* [7] and Pasko [8] that age-related upsurges in phenolic compound levels and antioxidant activities typically accompany an increase in mature plants’ capabilities to devote resources to secondary metabolic processes. In contrast, the more restricted resources of younger plants would be utilized more during primary metabolic processes that are needed for growth. Achakzai* et al.* [9] reported that minimum quantity of total phenolic was found in young leaves of *Nerium oleander*, while young leaves and stem of *Rhododendron* sp., contained maximum phenolic contents. As such, the flavonoid content detected in the leaves of young plants and shoots is greater than that in older plants. The medicinal properties of plants vary with respect to different age. Therefore, the authentic part of medicinal plants of a particular age should be harvested in a particular season before processing for drug manufacture, to avoid any alteration in its medicinal potency [1].

Sabah snake grass (*Clinacanthus nutans* L.) was originally found and is grown in tropical Asia. This plant is a well known anti-snake venom amongst the traditional healers of Thailand. Sabah snake grass is utilized in Malaysia as a traditional medicine, particularly for treating skin rashes, scorpion and insect bites. In China the whole plant is used in various manners to treat inflammatory conditions like haematoma, contusion, strains and sprains of injuries and rheumatism [10]. It can be also used for treating genital herpes and VZV lesions diagnosed in immunocompromised people [11]. Due to the numerous identified useful benefits of this crop, which is also cultivated in Malaysia, it is important to conduct further studies to determine its bioactive compounds and pharmaceutical properties. Currently, little is known about the secondary metabolites and the dynamic variation of these components during the growth period of sabah snake grass. It is important to gather relevent evidence on foods with high levels of these potentially beneficial components. To the best of our knowledge, no other studies have been undertaken to determine the alteration of bioactive compound synthesis and pharmaceutical quality of sabah snake grass at different plant growth periods. Current research is aimed at assessing changes in flavonoid and phenolic acid production in relation to chalcone synthase enzyme activity as well as examining their antioxidant and anticancer activities as a function of the growth period of sabah snake grass.

## 2. Results and Discussion

### 2.1. Changes in Total Flavonoid (TF) and Individual Flavonoid Concentration during the Plant Growth Period

All identified flavonoids showed good stability in their retention times. In addition, good repeatability within day and reproducibility between day in peak areas (RSD < 2%) were obtained. In general, repeatability and reproducibility were acceptable in all samples, indicating the precision in results obtained. Table 1 summarizes the experimental data on the flavonoid compounds of sabah snake grass. Significant differences (*p* < 0.05) were observed between the three plant growth periods and the different plant parts. Six-month-old buds had the highest content of TF (6.32 mg/g dried weight [DW]) followed by 1-year-old buds (5.42 mg/g DW) and 6-month-old leaves (4.66 mg/g DW). As shown in Table 1, the concentration of each flavonoid compound was significantly influenced by plant age. Catechin was identified in sabah snake grass, with its highest concentration found in 1-year-old plants. The amount of catechin was higher (2.023 mg/g DW) in buds compared to the leaves (1.191 mg/g DW) in 1-year-old plants; however, in 1- and 6-month-old plants the concentration of catechin was higher in the leaves compared to the buds. Kaempferol is a reportedly rare flavonoid in plants; however, in the current study, it was detected in the sabah snake grass extracts at reasonable concentrations. The highest levels of kaempferol were observed in 6-month-old buds (1.396 mg/g DW), followed by 1-year-old buds (0.910 mg/g DW). However, sabah snake grass extracts contain high levels of kaempferol in comparison with cekur manis (0.323 mg/g DW), pegaga (0.0205 mg/g DW), green chilli (0.039 mg/g DW), pumpkin (0.371 mg/g DW), and sengkuang (0.037 mg/g DW) [12].

The concentration of luteolin was influenced by plant age; the highest concentrations of this flavonoid were observed in 6-month-old buds (0.390 mg/g DW). Luteolin was not detected in 1- and 6-month-old leaves. As shown in Table 1, the highest concentration of quercetin was recorded in 6-month-old buds (1.669 mg/g DW), followed by 1-year-old buds (1.297 mg/g DW) and 1-year-old leaves (1.203 mg/g DW). In general, among the flavonoids isolated in this study, the important flavonoids based on concentration from high to low were as follows: catechin > quercetin > kaempferol > luteolin. The results indicate that catechin is abundant in sabah snake grass, which is an important consideration for future research. The results of the current study are inconsistent with previous reports that showed that flavonoid levels decrease during the growth period of plants [13,14]. The results of the current study are also inconsistent with those of Behn* et al.* [15] who reported the lowest quercetin content in the young leaves of red leaf lettuce compared to older leaves. The results of another study on ginger showed that flavonoid compounds decreased significantly in the leaf but increased in the rhizome during the growth period [16]. Fernandes *et al*. [17] compared the effect of harvesting time and soil composition on the synthesis of flavonoid compounds in *Calendula officinalis* and reported that the synthesis of five flavonoid compounds was influenced by the harvesting time, while no significant effect of soil composition was observed. On the basis of previous results and those of the current study, it is hypothesized that harvesting time could have the most impact on the synthesis of flavonoids in sabah snake grass.

**Table 1:** Total flavonoid content and some flavonoid compounds detected from sabah snake grass extracts.

Samples	TF	Catechin	Kaempferol	Luteolin	Quercetin
1-month-old buds	3.270 ± 0.331 ^d^	0.968 ± 0.014 ^e^	0.667 ± 0.060 ^c^	0.173 ± 0.046 ^b^	1.121 ± 0.007 ^b^
1-month-old leaves	3.790 ± 0.293 ^d^	1.244 ± 0.061 ^d^	0.475 ± 0.039 ^d^	ND	1.156 ± 0.189 ^b^
6-month-old buds	6.320 ± 0.740 ^a^	1.582 ± 0.072 ^c^	1.396 ± 0.236 ^a^	0.390 ± 0.179 ^a^	1.669 ± 0.451 ^a^
6-month-old leaves	4.660 ± 0.432 ^c^	1.711 ± 0.031 ^b^	0.855 ± 0.038 ^b^	ND	1.189 ± 0.099 ^b^
1-year-old buds	5.420 ± 0.408 ^b^	2.023 ± 0.125 ^a^	0.910 ± 0.034 ^b^	0.219 ± 0.153 ^b^	1.297 ± 0.187 ^b^
1-year-old leaves	4.120 ± 0.435 ^c^	1.191 ± 0.216 ^d^	0.632 ± 0.058 ^c^	0.115 ± 0.427^b^	1.203 ± 0.253 ^b^

Data are means of triplicate measurements ± standard deviation. Means not sharing a common single letter for each measurement were significantly different at *p* < 0.05. The units of all measurement are mg/g DW. ND: not detected.

### 2.2. Changes in Total Phenolic Acid (TP) and Individual Phenolic Acid Concentrations during the Plant Growth Period

As shown in Table 2, TP content was influenced by plant age and sample type. The range of TP content was between 6.840 and 18.210 mg/g DW. Six- and 1-month-old buds had the highest and lowest TP contents, respectively. Production of TP in 1-month-old plants was low, but then increased in 6-month-old plants (percentage of increasing in leaves: 55.2% and buds: 166%) before decreasing again in 1-year-old plants (percentage of decreasing in leaves: 15.1%; buds: 6.9%).

**Table 2:** Total phenolic content and some phenolic compounds detected from sabah snake grass extracts.

Samples	TP	Caffeic Acid	Gallic Acid
1-month-old buds	6.840 ± 0.470 ^d^	0.169 ± 0.034 ^b^	3.230 ± 0.645 ^b^
1-month-old leaves	7.290 ± 0.801 ^d^	0.180 ± 0.028 ^b^	2.931 ± 0.459 ^b^
6-month-old buds	18.210 ± 1.125 ^a^	0.204 ± 0.014 ^b^	5.963 ± 0.545 ^a^
6-month-old leaves	11.320 ± 1.280 ^c^	0.172 ± 0.033 ^b^	5.339 ± 0.468 ^a^
1-year-old buds	15.460 ± 1.231 ^b^	0.307 ± 0.018 ^a^	2.699 ± 0.380 ^b^
1-year-old leaves	10.530 ± 1.665 ^c^	0.282 ± 0.011 ^a^	2.166 ± 0.367 ^b^

Data are means of triplicate measurements ± standard deviation. Means not sharing a common single letter for each measurement were significantly different at *p* < 0.05. The unit of all measurement are mg/g DW.

Only at a young age (1-month-old) was the TP content in the leaves higher than in the buds; however, during the plant growth period, TP concentration was significantly enhanced in the buds. In the current study, two phenolic compounds were identified in the sabah snake grass extracts. Caffeic acid was detected in all extracts, and the highest content (0.307 mg/g DW) of this phenolic acid was detected in 1-year-old buds. Production of caffeic acid significantly (*p* < 0.05) increased from 1-month to 1-year-old plants. Gallic acid was also found at a reasonable concentration in sabah snake grass, varying between 2.166 mg/g DW in 1-year-old leaves to 5.963 mg/g DW in 6-month-old buds. Production of gallic acid, like TP, increased from 1 to 6 months of plant growth (leaves: 82%; buds: 84%); however, this significantly decreased from 6 months to 1 year of plant growth (leaves: 59%; buds: 54%). Romani* et al.* [18] suggest that caffeic acid content is higher in lettuce leaves in the early compared to later growth stages. Our results are consistent with previous studies that demonstrated that synthesis and accumulation of phenolic compounds is influenced by plant age [16,19,20].

### 2.3. The Enzyme Chalcone Synthase (CHS, EC 2.3.1.74) Activity

**Figure 1:**
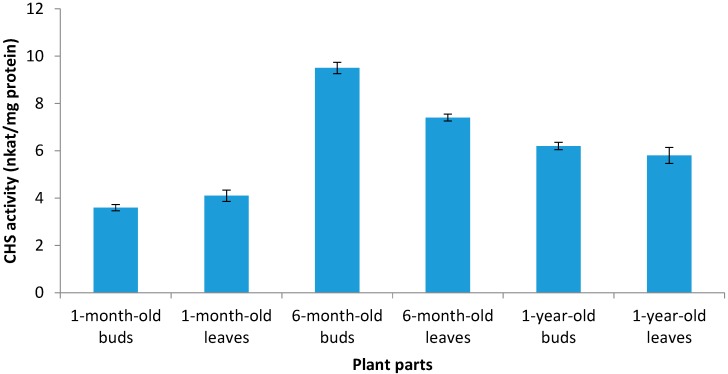
CHS activity in buds and leaves of sabah snake grass at different plant age. Error bars represent standard errors of the mean.

The enzyme chalcone synthase (CHS, EC 2.3.1.74) was discovered and reported as a key enzyme for flavonoid metabolism in plant cells [21]. As shown in Figure 1, CHS activity was significantly influenced by plant age. The lowest and highest activity levels of CHS were recorded in 1- and 6-month-old buds with values of 3.6 and 9.5 nkat/mg protein, respectively. From 1 to 6 months, CHS enzyme activity was enhanced in seedlings by about 80.4% and 163.8% in the leaves and buds, respectively. In contrast, with increasing growth period from 6 months to 1 year, CHS enzyme activity significantly decreased by about 21.6% and 34.7% in the leaves and buds, respectively. The mechanism of the CHS enzyme effect on flavonoid synthesis has been reported previously. CHS may always be present in the cells but is only activated under certain specific conditions means that CHS is activated at the protein level.

**Figure 2:**
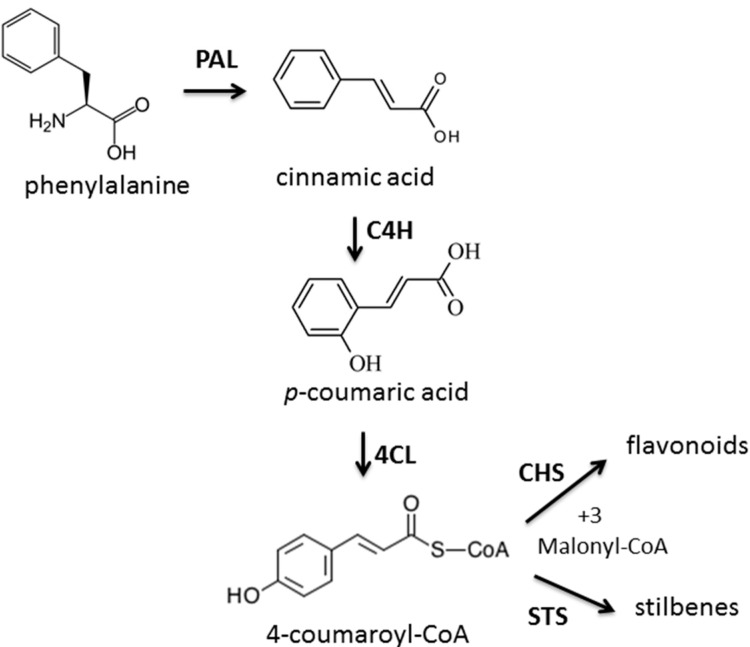
Mechanism of CHS enzyme on flavonoid synthesis [27].

As shown in Figure 2, flavonoids were derived from 4-coumaroyl-CoA and malonyl-CoA in the presence of the CHS enzyme. This indicates that the CHS enzyme is an important enzyme for flavonoid synthesis. According to the results from the current study, it is hypothesized that the increment of polyphenolic compounds in 6-month-old buds and leaves could be attributed to an increase in CHS activity. Ozeki* et al.* [22] reported that changes in anthocyanin accumulation were correlated with changes in CHS activity rather than phenylalanine ammonia-lyase (PAL) activity. With increasing plant age from 1 to 6 months, CHS activity increased in the leaves and buds, however, between 6 months and 1 year, CHS activity decreased. Chen et al [23] noted that CHS activity increased at all times of the year in the leaves of *Ginkgo biloba* L. In *Scutellaria baicalensis* CHS was increased between flowering and seed drop and decreased in withered period [24]. Furthermore, Nicholson and Hammerschmidt [25] reported that CHS could be considered as a biochemical marker for evaluating dynamic changes in flavonoid synthesis in plants. Saslowsky and Winkel [26] examined the subcellular location of CHS in Arabidopsis roots. They reported high levels of CHS enzyme was found in the epidermal and cortex cells of the elongation zone and the root tip, consistent with the accumulation of flavonoid endproducts at these sites. However, for the enzymes of the flavonoid pathway, several mechanisms may be involved. In general, the results of current study demonstrated that CHS activity was varied during growth period of sabah snake grass.

### 2.4. In Vitro Antioxidant Activity

#### 2.4.1. 1,1-Diphenyl-2-picrylhydrazyl (DPPH) Radical Scavenging Activity of Sabah Snake Grass during the Plant Growth Period

**Figure 3:**
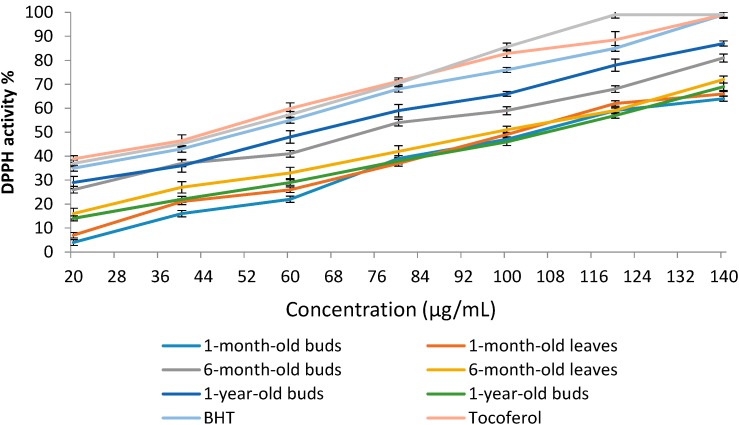
Free radical scavenging activity (DPPH assay) of the buds and leaves extract of sabah snake grass at different growth age. Error bars represent standard errors of the mean.

The DPPH radical scavenging activities of sabah snake grass leaf and bud extracts after different growth periods are shown in Figure 3. After different growth periods, bud extracts exhibited higher free radical scavenging activities compared with leaf extracts. The DPPH activities also significantly (*p* < 0.05) increased in the buds and leaves between 1 month and 1 year. The highest value of DPPH was observed in the extract of 1-year-old buds followed by 6-month-old buds. As shown in Figure 2, 50% of free radical scavenging (IC_50_) was observed in 1-year and 6-month-old buds at a concentration of 64.6 and 73.5 µg/mL, respectively. In a recent study, Yong* et al.* [28] reported that the IC_50_ of sabah snake grass extract for DPPH activity was observed at a concentration of 47.7 µg/mL. In another study, the maximum DPPH activity of sabah snake grass was reported as 67.65% with an IC_50_ of 110.4 μg/mL [11]. In the current study, all extracts showed an IC_50_ value at concentrations below 112.2 µg/mL. As shown in Table 3, the DPPH activities of the sabah snake grass extracts after different growth periods were less than those of butylated hydroxytoluene (BHT) (68.0%), caffeic acid (70.4%) and α-tocopherol (71.2%) at concentration of 100 µg/mL. At this concentration (100 µg/mL), 50% of extracts reached the IC_50_ value. The high DPPH activity in extracts from 6-month-old buds could be related to the higher content of phenolic compounds at this age. Pannangpetch* et al.* [29] reported the antioxidant activity and protective effects against oxidative hemolysis of sabah snake grass extract.

**Table 3:** DPPH scavenging activities and IC_50_ value of the buds and leaves extract of sabah snake grass at different growth age at concentration of 100 µg/mL.

Extracts	DPPH Activity %	IC_50 _(µg/mL)
1-month-old buds	47.4 ± 2.45 ^d^	110.4 ± 0.48 ^b^
1-month-old leaves	49.1 ± 2.19 ^d^	106.8 ± 0.82 ^c^
6-month-old buds	59.6 ± 1.87 ^b^	73.5 ± 0.45 ^e^
6-month-old leaves	51.3 ± 2.10 ^c^	98.2 ± 0.74 ^d^
1-year-old buds	66.2 ± 1.42 ^a^	64.6 ± 0.39 ^f^
1-year-old leaves	46.6 ± 2.77 ^d^	112.1 ± 0.58 ^a^
BHT	76.5 ± 1.92	52.6 ± 0.44
Tocoferol	82.8 ± 3.15	44.0 ± 0.61
Caffeic acid	85.6 ± 2.74	49.2 ± 0.85

Data are means of triplicate measurements ± standard deviation. Means not sharing a common single letter for each measurement were significantly different at *p <* 0.05.

#### 2.4.2. Ferric Reduction Antioxidant Potential (FRAP) Activity

**Figure 4:**
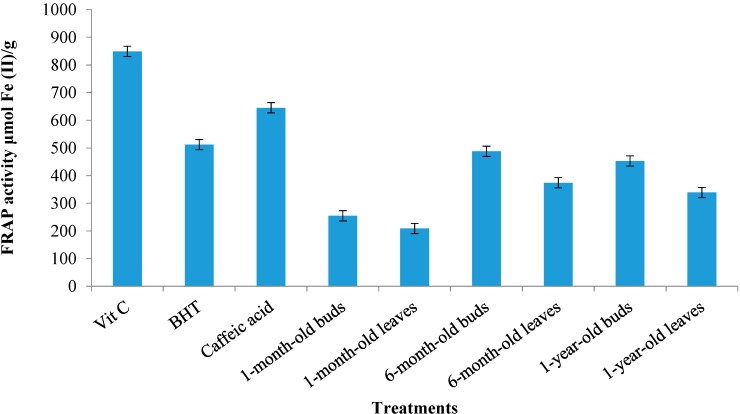
FRAP activity of sabah snake grass extracts during growth period. Error bars represent standard errors of the mean.

As shown in Figure 4 the FRAP activity of sabah snake grass extract ranged between 209 μM of Fe (II)/g (1-month-old leaves) and 488 μM of Fe (II)/g (6-month-old buds). The FRAP activities for all sabah snake grass extracts were significantly lower than those shown by the standard antioxidants, BHT and vitamin C (512.5 and 849.8 μM Fe (II)/g, respectively). Many previous studies have highlighted the potential role of flavonoids and phenolic acids from herbs and spices, which may act as antioxidants [30,31]. Interestingly, the results of the FRAP assay showed a higher activity in 6-month-old buds (488 μM of Fe (II)/g) than in 1-year-old buds (453 μM of Fe (II)/g), which conflicts with the DPPH result. According to the DPPH result, the free radical scavenging of 1-year-old buds was higher than that of 6-month-old buds. A possible explanation for this might be that in sabah snake grass extracts the FRAP assay favored flavonoid compounds compared to the DPPH assay. Future, studies on this topic are therefore recommended.

### 2.5. Correlation between Identified Polyphenolic Compounds and Antioxidant Activity

As shown in Table 4, significant (*p* < 0.05) correlations were found between DPPH and FRAP activity in the sabah snake grass extracts. TF content was significantly correlated with DPPH and FRAP activity; however, the correlation between TF and FRAP activity was stronger than that between TF and DPPH activity. Of the identified flavonoid compounds, only catechin showed a significant correlation with DPPH activity. In addition, kaempferol and quercetin displayed a significant correlation with FRAP activity. TP content also correlated significantly with DPPH (r^2^ = 0.811) and FRAP (r^2^ = 0.97) activity. Of the identified phenolic acids, gallic acid showed a significant correlation with DPPH and FRAP activity. As can be seen from the data, the polyphenols were correlated most strongly with FRAP activity. Results of certain previous studies [32,33,34] have demonstrated a significant correlation between flavonoids and phenolic acids and their antioxidant activity, while other studies [35,36] have shown poor correlation or report no significant correlation between polyphenol content and antioxidant activity. Significant correlations (*p* < 0.05) between CHS enzyme activity and FRAP activity, TF, catechin, and kaempferol content were observed. As shown in Table 4, no significant correlation was observed between CHS enzyme activity and TP, gallic acid and caffeic acid content.

**Table 4:** Correlation analysis between identified compounds and antioxidant activity of sabah snake grass extracts.

Variables	1	2	3	4	5	6	7	8	9	10	11
1 DPPH	1										
2 FRAP	0.777 *	1									
3 TF	0.802 *	0.932 **	1								
4 Catechin	0.875 *	0.748	0.748	1							
5 Kaempferol	0.644	0.883 *	0.907 *	0.535	1						
6 Luteolin	0.073	0.353	0.16	−0.202	0.217	1					
7 Quercetin	0.649	0.921 **	0.946 **	0.525	0.953 **	0.386	1				
8 TP	0.811 *	0.97 **	0.985 **	0.727	0.906 *	0.305	0.96 **	1			
9 Caffeic Acid	0.745	0.765	0.599	0.607	0.45	0.629	0.58	0.708	1		
10 Gallic Acid	0.828 *	0.996 **	0.594	0.341	0.761	−0.333	0.599	0.512	−0.165	1	
11 CHS	0.286	0.88 *	0.81 *	0.79 *	0.82 *	0.487	0.154	0.059	0.065	0.304	1

* and ** = significant at *p <* 0.05 and *p <* 0.01 respectively.

### 2.6. In Vitro Anticancer Activity

**Figure 5:**
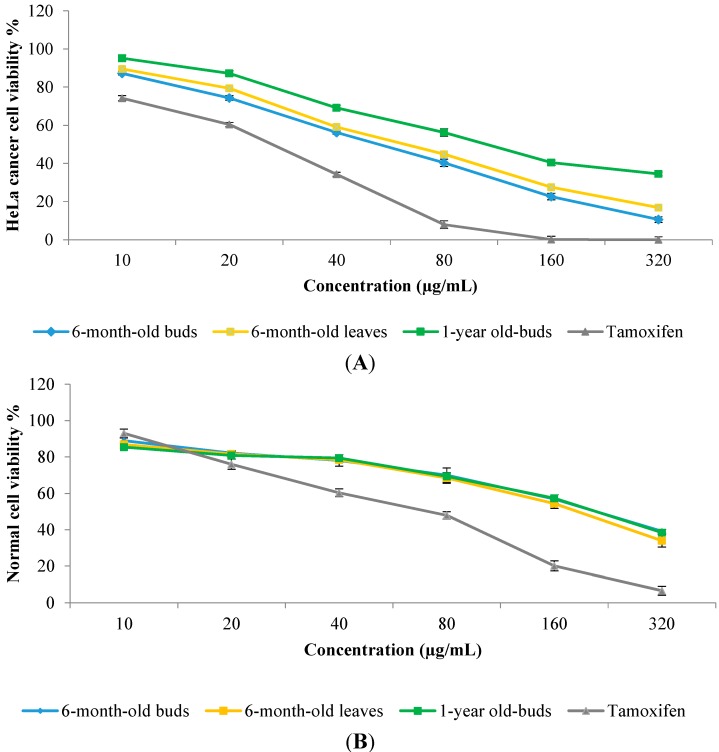
Dose-dependent anticancer activity of sabah snake grass extracts against HeLa cell line (**A**) and normal cell viability (**B**). Tamoxifen was used as a positive control. Error bars represent standard errors of the mean.

On the basis of the data for the secondary metabolites and antioxidant activity, the extracts of 6-month and 1-year-old buds and 6-month-old leaves of sabah snake grass were chosen for evaluation of their anticancer activity against the HeLa cancer cell line. Preliminary screening showed that sabah snake grass extracts exhibited a significant (*p* < 0.05) anticancer activity against HeLa cancer cells at a concentration of 56.8 µg/mL, with an inhibition rate of 50% with 6-month-old bud extract (Figure 5). HeLa cells were inhibited by 76.7% when treated with tamoxifen (positive control) at a concentration of 56.8 µg/mL. The 50% inhibitory concentration (IC_50_) value of the extracts of 6-month and 1-year-old buds and 6-month-old leaves of sabah snake grass were found at a concentration of 56.8, 110.4, and 64.3 µg/mL, respectively. According to the National Cancer Institute (NCI), a crude extract can be considered as active if it possesses an IC_50_ value of less than 20 μg/mL. However, in the current study the obtained IC_50_ values fell above that recommended by the NCI for crude extracts. That means is sabah snake grass is not suitable for anticancer drug discovery against HeLa cancer, but it could be used as a food crop for health-promoting properties. As shown in Figure 5, normal cells treated with the above concentrations (IC_50_) of sabah snake grass extract (6-month and 1-year-old buds and 6-month-old leaves) showed 78.5, 65.9, and 73.8% cell viability, respectively. Results showed all extracts to be non-toxic at concentrations below 240 µg/mL, but above that, toxic effects were evident. In a recent study, Yong* et al.* [28] reported antiproliferative effects of sabah snake grass extract against a human erythroleukemia cell line (K-562) with a value of 91.28% at a concentration of 100 µg/mL. In addition, it was reported that 1.8 g/kg of sabah snake grass extract was safely used in mice without causing mortality [37]. It has been demonstrated that the pharmaceutical qualities of herbs are related to bioactive compounds like alkaloids, phenolics, flavonoids,* etc.* [38,39,40]. The concentrations of these components vary during the growth period of plants, and it is therefore important to identify the best harvesting time in order to obtain the highest concentration of bioactive compounds. Based on obtain results, 6 months after plantation is the most suitable harvesting time to obtain sabah snake grass of high pharmaceutical quality. When comparing the different plant parts, the buds exhibited good pharmaceutical quality. To the best of our knowledge, this is the first report of alteration in the pharmaceutical quality of sabah snake grass during the plant growth period, and the results of the current study could be useful for future studies.

## 3. Experimental Section

### 3.1. Plant Material

A stem cutting of Sabah sabah snake grass was planted in a small polyethylene bag filled with humus. The germinated stems were grown for 2 weeks and transferred to larger polyethylene bags containing a mixture of soil, chicken manure, and burnt rice husk at a ratio of 3:1:1. The experiment was carried out under glasshouse conditions at the University Putra Malaysia (UPM). The daily temperature was 26–33 °C, relative humidity was 70%–80% and irradiance level was 44–1650 μmol/m^2^/s. The plants were harvested after various growth periods (1, 6 and 12 months), and then buds and leaves were separated and freeze dried. Once dried, they were stored at −80 °C for future analysis.

### 3.2. Extraction

Methanol (20 mL) was added to samples (0.25 g) and solutions were shaken gently at room temperature (25 °C) for 2 h. Homogenized samples were treated with 6 M HCl (5 mL) and refluxed at 70 °C for another 2 h. The solutions were cooled at room temperature and filtered through a 0.45 µm membrane for future analysis.

### 3.3. Determination of Total Phenolic Content

The content of total phenolics from the sabah snake grass was evaluated by the Folin-Ciocalteu method [41]. The hydrolyzed leaf and bud extracts (100 µL) were transferred to tubes and adjusted to 10 mL with distilled water. Then 500 µL of diluted Folin-Ciocalteu reagent was added to the solutions which were allowed to stand for 10 min at 25 °C. Later, saturated sodium carbonate (1000 µL, 20%) was added to the solutions and kept in total darkness at room temperature for 20 min. The absorbance of blue color was measured at 760 nm (UV2550, Shimadzu, Japan). Gallic acid was used as a standard for the calibration curve.

### 3.4. Determination of Total Flavonoids

Leaf and bud extract of sabah snake grass (1 mL) was added to 10-mL volumetric flasks containing 4 mL methanol. NaNO_2_ solution (1:5, w/v, 0.3 mL) was added to the flasks and allowed to stand for 6 min at room temperature. After 6 min, 0.3 mL AlCl_3_ solution (1:10, w/v) was added to the flasks, mixed well and kept for 6 min at room temperature. At last 2.0 mL NaOH solution (1 M) was added and kept for 10 min at room temperature. The absorbance was measured at 510 nm by spectrophotometer (UV2550, Shimadzu, Japan) [42]. Quercetin was used as a standard for the calibration curve.

### 3.5. Separation and Analysis of Flavonoids and Phenolic Acids by UHPLC

A UHPLC system (1290 Infinity Quaternary LC System, Agilent, Santa Clara, CA, USA) equipped with a C18 (5 μm, 4.6 × 250 mm) column was used for flavonoid separation and identification. In this system two mobile phase including: 0.03 M *ortho*-phosphoric acid (A) and methanol HPLC grade (B) were used. The column temperature, flow rate and injection volume were adjusted at 35 °C, 20 µL and 1 mL/min, respectively. The range of detecting wavelength was between 260–360 nm. Gradient elution was performed as follows: 0–10 min 40%–100% B; 10–15 min 100% B; 15–20 min 100%–40% B and finally, washing of the column. To prepare the standard solution (+)-catechin hydrate (≥98%, C1251 SIGMA, Petaling Jaya, Selangor, Malaysia), kaempferol (≥97%, 60010 SIGMA), luteoli 7-*O*-β-d-glucoside (≥98%, 74284 SIGMA), quercetin dehydrate (≥98%, Q0125 SIGMA), gallic acid monohydrate (≥99%, 27645 SIGMA) and caffeic acid (>98%, C0625 SIGMA) were dissolved in HPLC grade methanol with varied concentrations. The linear regression equation were calculated with Y = aX ± b, where X was concentration of flavonoid and Y was the peak area of flavonoids obtained from UHPLC. The linearity was established by the coefficient of determination (R^2^). Precision was evaluated by determining within-day variation of the UHPLC analysis and long term variation of the whole method over three consecutive weeks. Selected sample extract was analysed six times within a day to study for the repeatability.

### 3.6. Chalcone Synthase (CHS) Assay

The CHS was extracted from 0.4 g of plant samples with a solution of 1 mM 2-mercaptoethanol dissolved in 0.1 M borate buffer (1 mL, pH 8.8) at 4 °C. Subsequently, Dowex l × 4 resin (0.1 g) was added to the solution and the mixture rested for 10 min. The solution was then centrifuged at 15,000 rpm for 10 min to remove the resin. The supernatant was transferred to a tube, and Dowex resin (0.2 g) was added and the mixture left standing for 20 min. The resin was removed from solution after centrifugation at 15,000 rpm for 15 min. The supernatant (100 μL) was mixed gently with 10 mM potassium cyanide and following that Tris-HCI buffer (1.89 mL, pH 7.8) was added. Subsequently, chalcone (10 mg) was added to ethylene glycol monomethyl ether (10 μL), mixed with enzyme extract, and the reaction allowed to proceed for 1 min at 30 °C. The absorbance was measured at 370 nm (UV2550, Shimadzu, Japan).

### 3.7. Evaluation of in Vitro Antioxidant Activity

#### 3.7.1. 1,1-Diphenyl-2-picrylhydrazyl (DPPH) Assay

The free radical scavenging activity of extracts were determined according to the Hsu* et al.* [43] with some modifications. A 2.0 mL of hydrolyzed sabah snake grass extracts (dissolved in methanol) at various concentrations was mixed with 2.0 mL of 200 µM DPPH solution in methanol. The mixture was shaken gently and incubated at 28 °C in a dark room for 30 min. For the control, methanol was used as a blank. The absorbance of the samples was read at 517 nm using spectrophotometer (UV2550, Shimadzu, Japan). Butylated hydroxytoluene (BHT) caffeic acid and α-tocopherol, were used as positive controls. The scavenging activity was calculated using the following formula:

% inhibition = [(absorbance of control – absorbance of sample)/absorbance of control)] × 100
(1)

#### 3.7.2. Ferric Reducing Antioxidant Potential (FRAP) Assay

The FRAP assay was carried out according to the procedure of Dudonne *et al.* [44] with slight modification. FRAP stock solutions was prepared with mixing of 25 mL acetate buffer (30 mM, pH: 4), 2.5 mM 2,4,6-tripyridyl-S-triazine solution (TPTZ) in 40 mM HCl, 2.5 mL 20 mM FeCl_3_. The mixture was incubated at 37 °C for 15 min. for the analysis, 150 µL of sample extracts or standards was added to fresh FRAP solution (2.85 mL) and incubated for 30 min. The absorbance of solution was read at 593 nm (UV2550, Shimadzu, Japan). For a blank water (10 mL) was added to FRAP solution (300 mL). After 4 min the blank was zeroed and the absorbance of samples was recorded. The difference between blank and sample was used to calculate the FRAP value. The reducing power of the samples was determined from a calibration curve using different concentrations of BHT, caffeic acid and vitamin C.

### 3.8. Determination of in Vitro Anticancer Activity Using MTT Assay

#### 3.8.1. Extract Preparation

Dried leaves and buds (1 g) of sabah snake grass were powdered and extracted using methanol (50 mL) with continuous swirl for 1 h at 25 °C using an orbital shaker. Homogenized samples were refluxed at 40 °C for another 2 h. Extracts were filtered, evaporated and crude extract stored at −20 °C. These crude extracts were used for MTT assay.

#### 3.8.2. MTT Assay

The frozen HeLa cancer cells (human cervical adenocarcinoma) were retrieved from liquid nitrogen cell storage tank and rapidly thawed in cyrovials. The contents of the cyrovial were carefully transfered to a centrifuge tube and pre-warmed media (10 mL) was added slowly to the cell suspension. After spinning down at 1000 rpm for 10 min the pellet was gently resuspended in fresh media (10 mL) in a culture flask and incubate in a 37 °C humidified incubator supplemented with 5% CO_2_. After 24 h, the old medium was discarded and PBS (2–3 mL) was added to cover all the surface and then discarded. After adding trypsining solution (1.5–2 mL) to cover the flask surface the mixture was left at room temperature for 3 min until most of the cells detached. Complete medium (10 mL) was then added. For the MTT assay cells (100 μL) were added into all the wells with various concentrations of extracts (10, 20, 40, 80, 160 and 320 μg/mL) and incubated in a 37 °C, 5% CO_2_ incubator for 72 h. A stock solution of 5 mg/mL MTT was prepared in PBS and MTT reagent (20 μL) was added to the cell monolayer. During this period, living cells produced blue insoluble formazan from the yellow soluble MTT. The reaction was stopped by addition of Dimethyl sulfoxide (DMSO, 100 μL) to each well by pipetting 10–20 times to dissolve the blue formazan crystals. Control cells were incubated without the extract and with DMSO. The absorbance of samples was read at 540 nm using an ELISA reader (Biochrom, Holliston, Middlesex County, MA, USA). The absorbance of the formazan treated wells in the visible region correlates with the number of viable cells as follows:

Viable cells (%) = [(A_t_ − A_B_)/(A_C_ − A_B_)] × 100 (2)

where A_C_ is the absorbance of control, A_t_ is the absorbance of treated samples, and B is the absorbance of the blank.

### 3.9. Statistical Analysis

All data from the study were presented as mean ± SD of three replications, and means were compared using analysis of variance (ANOVA) by Statistical Analysis System (SAS 9.0, SAS Institute, Cary, CA, United States). Acquired data were manipulated to calculate statistical values such as mean and standard deviation (SD) using Microsoft Excel (Microsoft Inc., Redmond, WA, USA). The assumptions of normality and constant variance were tested using Anderson-Darling test and examining residual* versus* fit.

## 4. Conclusions

Our results showed dynamic changes in the content of flavonoids and phenolic acids in the buds and leaves of sabah snake grass as the plant growth period progressed. The extract of buds yielded the highest secondary metabolite content, antioxidant and anticancer activity, followed by the leaves. The results of current study suggested that CHS can be considered a biochemical marker for evaluating dynamic changes in flavonoid synthesis in plants. The results of our study showed that sabah snake grass is rich in flavonoids and demonstrated good free radical scavenging activity when measured by different methods. Therefore, for high pharmaceutical quality, sabah snake grass should be harvested 6 months after planting because of the high accumulation of flavonoids and phenolic acids at this growth stage, in particular in the buds. This might be due to increased translocation of the polyphenols into the buds from other parts of the plant or biosynthetic enzyme activities. Early harvesting of sabah snake grass leaves (six months), while they are slightly smaller than at conventional harvest time, may yield increased concentrations of secondary metabolites, which are potent antioxidant compounds. It is concluded that sabah snake grass is a good natural antioxidant.
